# Notes and News

**Published:** 1894-02-03

**Authors:** 


### Notes and News.

Collected and Communicated.

There are now no less than 20 women members of the British Medical Association, all of whom are fully qualified medical practitioners. At the last annual meeting of the British Association for the Advancement of Science, it was decided that the medical section of the society should be thrown open to feminine membership. This resolution was seconded by Mrs. Garrett Anderson, who was herself a member before 1876.

We have been asked to state that the Sub-committee appointed by the Lancashire Asylums Board on the 22nd ult. to select a successor to Dr. Wallis as medical superintendent of Whittingham Asylum met on the 29th ult., but owing to the limited number of applicants who presented themselves, they decided to postpone for a fortnight the filling up of the vacancy. This deadlock is caused by the non-insertion of an advertisement announcing the vacancy, and may show a remarkable lack of business knowledge on the part of those immediately responsible; or is it due to an attempt to run a particular candidate by a few of the Election Committee, as such avoidance of publicity usually means wire-pulling or design somewhere.

Our article on "Hospital Abuse and the Working Classes" has attracted considerable attention in Leeds, and has been reproduced in full in the *Leeds Mercury* and other papers. It is causing a great deal of discussion locally, and we have reason to hope that it may lead to the trial of a new and improved system of hospital relief in connection with the General Infirmary and all the other medical institutions in Leeds. It is fortunate that the consideration of the important questions involved will have the cordial co-operation of the Chairman of the Infirmary, Mr. R. Benson Jowett, and of Mr. Alderman Spark, whose exceptional experience and grasp of the subject must tend to facilitate matters immensely. We shall watch the result with genuine interest and hopefulness.

At a recent meeting of the managers of the Edinburgh Royal Infirmary "the question of active members of the staff occupying seats on the infirmary board was discussed." Very strong opinions were expressed by several members of the board against the appointment of Dr. Argyll Robertson and Dr. John Duncan as managers, and it was submitted that if they were to remain on the board they could not be continued as members of the staff. Letters were also read from Dr. Argyll Robertson and Dr. Duncan protesting against their appointment to the staff being made interim, and a letter was also read from Mr. Haldane, chairman of the Committee of Contributors, expressing strong disapproval of the appointment as managers of any member of the infirmary staff, and the whole matter was ultimately remitted to the Law Committee which reported the election to be technically in order. On the motion of the Lord Provost, seconded by Bailie M'Donald, the meetings of the managers will in future be open to the press.

We have received the audited balance-sheet of the Hospital Saturday Fund Cycling and Athletic Sports Committee. The total receipts were £729 17s. 7d., to which must be added £20 4s. 6d. for sums received since the accounts were closed. The expenses amounted to £383 3s. 10d., and only £300 was paid to the credit of the Hospital Saturday Fund. Whilst fully recognising the good intentions and kindly spirit which has animated the committee charged with the arrangements, we are convinced that an outlay of over one hundred per cent. is unjustifiable and calculated to do more harm than good to the object which these sports were avowedly instituted to promote. It is proposed to hold one meeting during 1894 at Herne Hill on May 26th, when, should the results prove equally unsatisfactory, we hope the movement will be discontinued in future years. The donations and prizes amounted altogether to £263 11s. 4d., a fact which eloquently endorses the criticisms we have felt it our duty to make in regard to these sports.

A meeting of the Belfast Royal Hospital was recently called to consider three proposals: (1) The scheme for the purchase of the premises of the Charitable Society, (2) the offer of the adjoining ground belonging to the Society of Friends, and (3) the extension of the out-patients' department on the present premises. It has been evident for some time past that the growth of the city of Belfast necessitates a like extension of the General Hospital charged with the care of its sick poor. Granting the possibility of raising the necessary funds—respecting which several present at the meeting appear to be far from hopeful—there then arose the question of an entirely new hospital or an extension on the present site, with which the two first proposals before the meeting dealt. Having regard to the fact that the present site is acknowledged to be far from an ideal one, and the air space already limited, the corporation of the hospital passed a resolution in favour of a scheme for a new hospital on a new site, but left the decision as to the exact locality for further consideration and suggestion. The immediate extension of the out-patient department met with unquestioning approval.

